# Peer Review of “Automating Individualized Notification of Drug Recalls to Patients: Complex Challenges and Qualitative Evaluation”

**DOI:** 10.2196/82613

**Published:** 2026-01-13

**Authors:** Alissa Russ

**Affiliations:** 1 Purdue University, 575 Stadium Mall Drive, West Lafayette, IN, United States, 1 765-494-1469

**Keywords:** notification system, drug recalls, patient safety, medication, electronic health records, prescriptions, decision support

*This is a peer review report for “Automating Individualized Notification of Drug Recalls to Patients: Complex Challenges and Qualitative Evaluation.”*

## Round 1 Review

### General Comments

This manuscript [1] describes interesting and novel work with far-reaching patient safety implications. The authors developed an automated system in the electronic health record (EHR) of an academic medical center that scans for drug recalls, matches up National Drug Codes of recalled medication on a patient’s medical list, and sends notifications through the EHR portal to the patient, providing them with more information on the recall. The authors then conducted a qualitative analysis of 9 patients’ perceptions of a fictious recall notice. Despite successful development of the automated system, many limitations prevented the widescale adoption of this system in 2 clinics associated with the large academic medical center. The outcome of the work—a decision was made not to deploy the new software for drug recalls—was surprising, and it is important that “failed” implementation work also be published. That said, key weaknesses of the manuscript are the lack of important details, need for better organization of the content, and the need for much stronger scientific and technical writing to accurately interpret the methods, results, and implications. These weaknesses also made it much more difficult to read and evaluate the manuscript. Despite the importance of the topic, the small sample size of patients also limits the work’s impact.

### Specific Comments

#### Title

It would be helpful if the title were a bit more specific about the technology, study methods (qualitative), and notification recipients (patients, providers, etc).

#### Abstract

1. The Background section appears to be contradictory. Sentence 2 says the Food and Drug Administration has ways to notify health care professionals (HCPs) and patients, but then the following sentences seem to say the opposite.
2. A few more details here on the type of platform would be helpful…software app? Web-based platform, etc? And what are the intended user types? (HCPs and patients? Or just patients?)
3. The choice of methods doesn’t seem to follow the Background section. Why was it necessary to include the clinics, rather than just work directly with the patients? Or, why was the focus on clinics, rather than pharmacies? (These comments apply to the main Introduction and Methods sections, as well.)
4. I expected the “program description” to appear in the Methods section, not the Results.

#### Introduction

1. The second and third sentences of the first paragraph of the Introduction: any studies or references to back up this claim?
2. No information is included on if/what literature explores this or similar topics.
3. I would recommend adding more information on the process pharmacies currently have in place for notifying patients of recalls. Also add any literature that exists showing how often patients then contact their providers or add quantitative data to highlight this extra burden on providers to emphasize the problem.
4. I expected the funding information in the last sentence of the first paragraph to be included in a funding statement or the acknowledgments (rather than the Introduction) and the rest of that statement to be described in the Methods.

#### Setting

1. I expected this to appear under a larger Methods section.
2. What was the goal sample size and rationale for the sample size? There is missing demographic information on the participating patients.
3. So the Fast Healthcare Interoperability Resources (FHIR) portion notified HCPs? The intended recipients are not specified for that part of the program.
4. “EHR build” was unexpected as a reader. Is that a third part? How does it fit into the first 2 parts?
5. The screenshots and figures are useful.
6. Even for a convenience sample, more details are needed on recruitment. How did you choose which patients to email? How many were emailed for recruitment? Were patients emailed and recruited sequentially, for example? Were there any exclusion or inclusion criteria for patients? Did any patients decline to participate? Why? What was the distribution of patients recruited from primary care versus cardiology?
7. More specific details are warranted for the methods used for qualitative analysis, such as whether an inductive versus a deductive design was used. Was a consensus approach used, or some other approach? See also the writing guidelines for qualitative studies (eg, the Consolidated Criteria for Reporting Qualitative Research [COREQ], Standards for Reporting Qualitative Research [SRQR]). Explain also the “additional verification” process during analysis. References should be cited for the qualitative methods used in this work.
8. Did any of the patients have prior experience with MyChart, and if so, what was the average number of years of MyChart experience?
9. These statements from the text appear to be contradictory, and the meaning of the first statement especially is unclear, and seems like an opinion: “[Patients expressed that the] widget should not ask patients to discuss the information with their healthcare provider.” “Patients wanted to discuss the recall with their clinicians to ‘close the loop.’”

1. The conclusion not to deploy the system seems dramatic based on the findings and makes me wonder if any other creative solutions were considered to address the concern of potential increased clinic burden. Also, how was it determined that the clinic burden outweighed safety risks to the patient? Maybe the system should only be used for certain types of recalls, for example. Or maybe the system could be integrated more with the pharmacy, rather than the prescriber’s clinic, or the letter could read differently (advising against contacting the clinic unless the patient was unable to resolve the issue with the pharmacy). Or the letter could explain that only the pharmacy, not the clinic, would have a record of the patient’s specific manufacturer and whether the recall applied to them.
2. It would be helpful to see the full interview guide and patient scenario details in a supplementary appendix to aid interpretation of the methods and results.

#### Discussion

1. The Discussion does not mention limitations of the study design and methods.
2. I expected at least some comparison to other, related literature.
3. Is anything stamped on the medication (eg, pill) itself to indicate the manufacturer? Or is that also inconsistent across medications?
4. A table of key recommendations could strengthen the paper.
5. In the last paragraph of the Discussion, there is no citation for the number of state boards of pharmacy that require the lot number to appear on the label.
6. I expected the Discussion to close with a Conclusions paragraph outlining key lessons learned and any generalizable findings.

## Round 2 Review

### General Comments

The authors addressed a few of my review comments and made some text changes, but unfortunately, most of my comments—about 15 of them—remain inadequately addressed. For the comments listed again below, the authors did not appear to change anything in the manuscript to address the comment. In many cases, even the authors’ reply to the reviewers did not answer the question. Also, the authors describe adding the interview guide as an appendix, but I could not find this file on the reviewer website.

Unaddressed or inadequately addressed review comments are described in the following sections.

### Specific Comments

#### Abstract

1. The Background section appears to be contradictory. Sentence 2 says the Food and Drug Administration has ways to notify HCPs and patients, but then the following sentences seem to say the opposite.

3. The choice of methods doesn’t seem to follow the Background section. Why was it necessary to include the clinics, rather than just work directly with the patients? Or, why was the focus on clinics, rather than pharmacies? (These comments apply to the main Introduction and Methods sections, as well.)

#### Introduction

2. No information is included on if/what literature explores this or similar topics. (Lack of literature citations/review.)

#### Setting

2. What was the goal sample size and rationale for the sample size? There is missing demographic information on the participating patients.

3. So the FHIR portion notified HCPs? The intended recipients are not specified for that part of the program.

6. Even for a convenience sample, more details are needed on recruitment. How did you choose which patients to email? How many were emailed for recruitment? Were patients emailed and recruited sequentially, for example? Were there any exclusion or inclusion criteria for patients? Did any patients decline to participate? Why? What was the distribution of patients recruited from primary care versus cardiology?

7. More specific details are warranted for the methods used for qualitative analysis, such as whether an inductive versus a deductive design was used. Was a consensus approach used, or some other approach? See also the writing guidelines for qualitative studies (eg, the COREQ, SRQR). Explain also the “additional verification” process during analysis. References should be cited for the qualitative methods used in this work.

8. Did any of the patients have prior experience with MyChart, and if so, what was the average number of years of MyChart experience?

9. These statements from the text appear to be contradictory, and the meaning of the first statement especially is unclear, and seems like an opinion: “[Patients expressed that the] widget should not ask patients to discuss the information with their healthcare provider.” “Patients wanted to discuss the recall with their clinicians to ‘close the loop.’”

10. The conclusion not to deploy the system seems dramatic based on the findings and makes me wonder if any other creative solutions were considered to address the concern of potential increased clinic burden. Also, how was it determined that the clinic burden outweighed safety risks to the patient? Maybe the system should only be used for certain types of recalls, for example. Or maybe the system could be integrated more with the pharmacy, rather than the prescriber’s clinic, or the letter could read differently (advising against contacting the clinic unless the patient was unable to resolve the issue with the pharmacy). Or the letter could explain that only the pharmacy, not the clinic, would have a record of the patient’s specific manufacturer and whether the recall applied to them.

#### Discussion

1. The Discussion does not mention limitations of the study design and methods.

2. I expected at least some comparison to other, related literature.

3. Is anything stamped on the medication (eg, pill) itself to indicate the manufacturer? Or is that also inconsistent across medications?

4. A table of key recommendations could strengthen the paper.

5. In the last paragraph of discussion, there is no citation for the number of state boards of pharmacy the require the lot number to appear on the label. (The statement that needs a literature citation is “Only three State Boards of Pharmacy require the NDC to appear on the dispensed medication label, and only five State Boards of Pharmacy require the lot number to appear on the dispensed medication label.”)

## Abbreviations

COREQ: Consolidated Criteria for Reporting Qualitative Research
EHR: electronic health record
FHIR: Fast Healthcare Interoperability Resources
HCP: health care professional
SRQR: Standards for Reporting Qualitative Research

## References

[1] Gadgil M, Pavlakos R, Carini S (2026). Automating individualized notification of drug recalls to patients: complex challenges and qualitative evaluation. JMIRx Med.

